# Pericarditis in Systemic Lupus Erythematosus: A Comprehensive Review of Pathogenesis, Diagnosis, and Management

**DOI:** 10.7759/cureus.112909

**Published:** 2026-07-18

**Authors:** Juan Camilo Santacruz, Marta Juliana Mantilla, Sandra Pulido, Carlos Agudelo, Julián Alberto Naranjo, Oscar Vicente Vergara, John Londono

**Affiliations:** 1 Department of Rheumatology, Medicarte IPS, Rionegro, COL; 2 Department of Rheumatology, Centro de Investigación en Reumatología y Especialidades Médicas (CIREEM), Bogotá, COL; 3 Department of Rheumatology, Colsanitas, Bogotá, COL; 4 Department of Rheumatology, Clínica Las Américas, Medellín, COL; 5 Department of Rheumatology, ART Médica IPS, Medellín, COL; 6 Spondyloarthritis Research Group, Universidad de La Sabana, Chía, COL

**Keywords:** biologic therapy, colchicine, pericardial effusion, pericarditis, serositis, systemic lupus erythematosus

## Abstract

Pericarditis is the most common cardiovascular manifestation of systemic lupus erythematosus and affects a substantial proportion of patients during the course of the disease. Although it is often mild and self-limiting, lupus-associated pericarditis can result in substantial morbidity due to recurrent episodes and potentially life-threatening complications, including cardiac tamponade and constrictive pericarditis. Recent advances have expanded our understanding of its immunopathogenesis, highlighting the interplay between immune complex deposition, complement activation, type I interferon signaling, and dysregulated cell death pathways. Diagnostic evaluation relies on clinical assessment supported by electrocardiography and transthoracic echocardiography, while cardiac MRI has emerged as a valuable modality for detecting active inflammation, characterizing pericardial tissue involvement, and identifying constrictive physiology. In contrast to idiopathic pericarditis, management strategies should address both pericardial inflammation and the underlying autoimmune disease activity. Colchicine has become a cornerstone of therapy owing to its effectiveness in symptom control and recurrence prevention, whereas glucocorticoids and immunosuppressive agents remain essential for selected patients with active systemic disease. Emerging targeted therapies, including anifrolumab, belimumab, and IL-1 inhibitors, offer promising alternatives for refractory or recurrent cases, although disease-specific evidence remains limited. This review provides a comprehensive overview of the current understanding of lupus-associated pericarditis, encompassing its pathophysiology, clinical manifestations, diagnostic approach, and therapeutic management, while highlighting recent advances and key areas for future research.

## Introduction and background

Cardiac involvement is common and clinically heterogeneous in patients with systemic lupus erythematosus (SLE), affecting the myocardium, pericardium, cardiac conduction system, heart valves, and coronary circulation. Pericarditis, with or without pericardial effusion, is the most frequent cardiovascular manifestation, occurring in approximately 25% of patients at some point during the disease course [1]. Pericardial involvement is often asymptomatic and may be detected incidentally during echocardiographic evaluation performed for other cardiovascular indications. Similar to other forms of serositis, lupus pericarditis typically occurs in the setting of active disease and is frequently accompanied by severe organ manifestations, particularly lupus nephritis [2]. Symptomatic pericarditis usually presents with moderate substernal chest pain and a characteristic pericardial friction rub best heard along the left sternal border. The presence of pericardial effusion and associated serosal involvement should be carefully assessed, as pericardial disease may lead to potentially life-threatening complications, including cardiac tamponade and constrictive pericarditis [3,4].

The reported prevalence of pericarditis in SLE ranges from 16% to 23% across different cohorts, with a median onset of approximately six months after diagnosis. Pericarditis appears to be more common among patients diagnosed at older ages and has been associated with anti-La antibodies, constitutional symptoms, lymphopenia, thrombocytopenia, and antiphospholipid antibody positivity [5]. In a cohort of 2,122 patients, pericarditis coexisted with myocarditis in 5.3% of cases and endocarditis in 1.9%, while cardiac tamponade occurred in 1.4%. Although most episodes resolved within three months, chronic pericarditis developed in 15.6% of patients, and clinical relapses were observed in 22.9% [6]. Similarly, among 590 patients with a history of lupus pericarditis, 20.3% experienced recurrent disease. Younger age at disease onset, higher SLE activity, positive anti-double-stranded DNA antibodies, oral prednisone use, and a shorter interval since the initial episode, particularly within the first year, were identified as major predictors of recurrence [7]. Vitamin D deficiency has also been associated with increased SLE activity and a higher frequency of serosal manifestations, including pericarditis, suggesting a potential immunomodulatory role in pericardial inflammation [8].

Electrocardiography may demonstrate the classic four-stage evolution of acute pericarditis, whereas transthoracic echocardiography (TTE) remains the cornerstone imaging modality for evaluating pericardial effusion, cardiac tamponade, and associated myocardial involvement [9]. In addition, cardiac MRI may provide valuable diagnostic information when the diagnosis remains uncertain or when myopericarditis, constrictive pericarditis, or complex pericardial effusions are suspected [10].

## Review

Pathophysiology

Lupus-associated pericarditis arises from a complex interplay between innate and adaptive immune responses, characterized by immune complex deposition, complement activation, autoantibody production, and the sustained release of proinflammatory cytokines. This immunopathogenic profile differs substantially from that of recurrent idiopathic pericarditis, in which autoinflammatory mechanisms driven predominantly by innate immune activation, NLRP3 inflammasome signaling, and IL-1β release play a central role [11].

A hallmark of SLE is the defective clearance of apoptotic material, which promotes the accumulation of nuclear autoantigens and perpetuates chronic immune activation. Genetic susceptibility factors contribute to this process. In particular, variants affecting complement receptor 3, such as the rs1143679 polymorphism in the ITGAM gene, impair the recognition and clearance of complement-opsonized immune complexes, facilitating the generation of anti-nucleic acid autoantibodies and increasing the risk of multisystem organ involvement [12].

Recent evidence has further highlighted the contribution of dysregulated programmed cell death pathways-including apoptosis, pyroptosis, necroptosis, neutrophil extracellular trap formation, and ferroptosis-to the immunopathology of SLE. The complex crosstalk among these mechanisms promotes persistent inflammation, autoantigen exposure, and tissue injury and has been increasingly implicated in the development of cardiovascular manifestations, including pericardial disease [13].

Activated plasmacytoid dendritic cells represent a major source of type I interferons (IFNs), particularly IFN-α, following stimulation by DNA- and RNA-containing immune complexes or apoptotic debris. The resulting IFN signature amplifies autoreactive B- and T-cell responses, enhances autoantibody production, and sustains systemic immune dysregulation. Simultaneously, macrophages exhibit impaired efferocytosis and defective clearance of apoptotic cells, contributing to the persistence of autoantigens and the release of proinflammatory mediators such as IL-1, IL-6, and type I IFNs [14].

At the tissue level, immune-mediated injury triggers a cascade of pericardial inflammation characterized by increased vascular permeability, exudative fluid accumulation, mesothelial cell desquamation, fibroblast proliferation, granulation tissue formation, and neovascularization. Persistent or recurrent inflammation may ultimately result in pericardial fibrosis, recurrent pericarditis, or progression to constrictive pericarditis, highlighting the importance of early recognition and effective control of the underlying autoimmune process [15].

**Figure 1:**
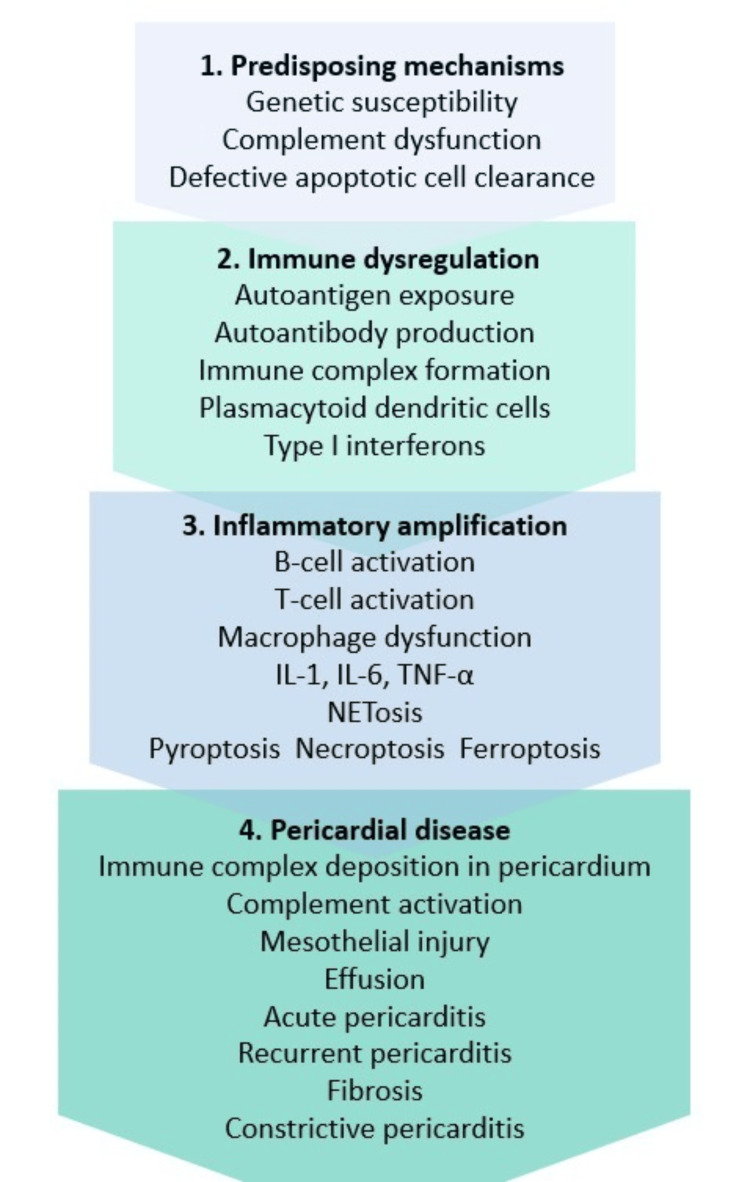
Proposed immunopathogenic cascade underlying lupus pericardial disease NETosis: neutrophil extracellular trap formation; TNF-α: tumor necrosis factor-alpha This figure was created using Microsoft PowerPoint (Microsoft Corporation, Redmond, WA, USA).

Figure 1 summarizes the proposed immunopathogenic cascade underlying lupus pericardial disease, illustrating the progression from genetic predisposition and immune dysregulation to inflammatory amplification and the development of pericardial inflammation and fibrosis.

Clinical manifestations

Acute pericarditis may present with a broad spectrum of clinical manifestations, although chest pain remains its hallmark symptom. More than 95% of patients with symptomatic pericarditis experience acute pleuritic chest pain, typically localized to the anterior chest and exacerbated by coughing, deep inspiration, or changes in position [16]. A distinctive feature of pericardial pain is its moderate-to-severe intensity, often improving when the patient sits upright and leans forward. This positional relief is thought to result from reduced mechanical stress on the inflamed parietal pericardium during respiratory movements and diaphragmatic excursion [17].

The characteristic pericardial friction rub is a highly specific physical finding caused by the movement of inflamed pericardial layers during the cardiac cycle. Classically, it consists of three audible components corresponding to atrial systole, ventricular systole, and early ventricular diastolic filling. In a study of 100 patients evaluated by both auscultation and phonocardiography, a triphasic friction rub was identified in 52% of cases, whereas 33% demonstrated a biphasic pattern and 15% exhibited only a single audible component [18].

Electrocardiographic abnormalities evolve through a characteristic sequence that reflects the underlying inflammatory process. Classical findings include diffuse ST-segment elevation and PR-segment depression during the acute phase, followed by normalization of these segments, subsequent T-wave inversion, and eventual restoration of a normal electrocardiographic pattern. Although this four-stage progression is considered typical, not all patients exhibit every stage, and atypical presentations are common in clinical practice [19].

Cardiac tamponade represents one of the most severe complications of acute pericarditis and requires prompt recognition and intervention. Despite the high prevalence of pericardial involvement in SLE, progression to cardiac tamponade or constrictive pericarditis remains relatively uncommon, occurring in approximately 2% of patients with lupus-associated pericarditis. Nevertheless, these complications are associated with substantial morbidity and may be life-threatening if left untreated [20].

Chest radiography has limited sensitivity for the diagnosis of pericarditis and is frequently normal in patients with small or moderate pericardial effusions. However, in cases of large effusions, radiographic findings may include enlargement of the cardiac silhouette with the classic “water-bottle” configuration, reflecting significant pericardial fluid accumulation [20].

Diagnostic imaging

TTE remains the first-line imaging modality for the evaluation of pericardial involvement because of its widespread availability, noninvasive nature, and cost-effectiveness. Beyond confirming the presence of pericardial effusion, echocardiography provides essential information regarding hemodynamic consequences and ventricular function. Effusion size is commonly estimated by the end-diastolic echo-free space and classified as trivial (visible only during systole), small (<1 cm), moderate (1-2 cm), large (>2 cm), or very large (>2.5 cm) [21,22].

Echocardiography also plays a pivotal role in the diagnosis of constrictive pericarditis by identifying the characteristic features of ventricular interdependence and dissociation between intrathoracic and intracardiac pressures. Key echocardiographic findings include respirophasic interventricular septal shift, inferior vena cava dilation with reduced inspiratory collapse, and increased medial mitral annular e′ velocity. The combination of these findings has been reported to provide a positive predictive value approaching 99% for the diagnosis of constrictive pericarditis [23].

Advanced echocardiographic techniques, including three-dimensional imaging and myocardial strain analysis, may further improve diagnostic accuracy. Patients with constrictive pericarditis frequently demonstrate a predominant reduction in circumferential strain and the characteristic annulus reversus pattern, in which septal longitudinal deformation exceeds lateral wall deformation as a consequence of mechanical restraint imposed by the thickened pericardium [24].

Cardiovascular magnetic resonance (CMR) has emerged as the most comprehensive noninvasive imaging modality for the characterization of pericardial disease and is particularly valuable when diagnostic uncertainty persists after echocardiographic evaluation. CMR allows simultaneous assessment of cardiac anatomy, ventricular function, pericardial thickness, and constrictive physiology through dynamic imaging sequences. In addition, T2-weighted short tau inversion recovery sequences enable the detection of pericardial edema, whereas late gadolinium enhancement (LGE) reflects increased gadolinium distribution within inflamed or fibrotic pericardial tissue, serving as a sensitive imaging marker of pericardial involvement that should be interpreted in conjunction with other CMR findings when assessing active inflammation [25].

In acute pericarditis, CMR typically demonstrates pericardial edema and inflammation through increased T2 signal intensity and LGE. As the disease progresses toward chronic stages, edema generally resolves, whereas persistent LGE may indicate ongoing inflammation and has been associated with an increased risk of recurrence [26]. Consequently, CMR has become an important tool not only for diagnosis but also for risk stratification and therapeutic decision-making.

In recent years, ¹⁸F-fluorodeoxyglucose PET/CT (¹⁸F-FDG PET/CT) has gained increasing attention as a functional imaging modality for the assessment of systemic inflammatory disorders. Its clinical utility is best established in conditions such as large-vessel vasculitis and sarcoidosis [27]. In patients with pericardial disease, PET/CT may provide complementary information regarding the underlying etiology. Higher levels of metabolic activity, typically reflected by a maximum standardized uptake value greater than 5, have been associated with tuberculosis, malignancy, and autoimmune-mediated pericarditis, whereas lower uptake values are more frequently observed in idiopathic disease [28].

The diagnostic performance of ¹⁸F-FDG PET/CT may be reduced by prior exposure to glucocorticoids, which suppress inflammatory metabolic activity and decrease tracer uptake. For this reason, temporary corticosteroid withdrawal should be considered whenever clinically feasible before imaging. Although PET/CT is not routinely required for the evaluation of lupus-associated pericarditis, it may be useful in selected cases when the etiology remains uncertain or when alternative inflammatory, infectious, or neoplastic causes are being considered [29,30].

Treatment

The management of lupus-associated pericarditis differs substantially from that of idiopathic pericarditis and remains largely guided by expert opinion and observational studies rather than randomized controlled trials. Consequently, no universally accepted treatment algorithm currently exists, and therapeutic decisions should be individualized according to disease severity, recurrence risk, and the degree of underlying SLE activity [31].

Because pericarditis frequently represents a manifestation of active SLE, treatment should aim not only to control pericardial inflammation but also to suppress the underlying autoimmune process. Colchicine has emerged as a cornerstone of therapy and is increasingly favored as first-line treatment, typically administered at a dose of 0.5-0.6 mg twice daily for approximately six months [32]. In a case series of 10 patients with lupus-associated pericarditis, colchicine achieved symptom resolution within a median of 2.5 days and prevented glucocorticoid initiation or dose escalation in most patients. Furthermore, continued or reintroduced colchicine successfully prevented recurrences during follow-up [33].

Although systemic glucocorticoids remain an important therapeutic option, particularly in patients with active multisystem disease, their use has been associated with an increased risk of recurrent pericarditis. In a retrospective cohort of 590 patients with lupus pericarditis followed for a median of 6.7 years, 20.3% developed recurrent disease. Prednisone doses exceeding 20 mg/day were associated with approximately a twofold increase in recurrence risk, highlighting the importance of minimizing corticosteroid exposure whenever clinically feasible [34].

Patients with recurrent, refractory, or steroid-dependent pericarditis frequently require additional immunosuppressive therapy. Azathioprine and mycophenolate mofetil are commonly employed as steroid-sparing agents, particularly in patients with concomitant systemic disease activity. Increasing clinical experience also supports the use of biologic therapies in selected cases [35].

Anifrolumab, a monoclonal antibody targeting the type I IFN receptor, has recently emerged as a promising therapeutic option. Although disease-specific evidence remains limited, a reported case of a 30-year-old woman with refractory lupus pericarditis demonstrated rapid clinical improvement, resolution of pericardial effusion, and normalization of inflammatory markers following initiation of anifrolumab at a dose of 300 mg intravenously every four weeks after inadequate response to glucocorticoids [36]. Given the central role of type I IFN signaling in SLE pathogenesis, this therapeutic approach is biologically plausible and warrants further investigation.

Similarly, evidence supporting the use of belimumab in lupus-associated pericarditis is restricted primarily to case reports. One notable case involved a 51-year-old woman with longstanding SLE and recurrent steroid-dependent pleuropericarditis despite treatment with hydroxychloroquine, methotrexate, and leflunomide. Following initiation of IV belimumab (10 mg/kg), the patient experienced complete resolution of pleural and pericardial inflammation, normalization of acute-phase reactants, successful glucocorticoid tapering, and sustained remission without further recurrences. These observations suggest that B-lymphocyte stimulator inhibition may represent an effective strategy in patients with refractory or steroid-dependent lupus serositis [37].

IL-1 inhibitors, including anakinra, rilonacept, and canakinumab, have revolutionized the treatment of recurrent idiopathic pericarditis. Although their role in SLE remains poorly defined and formal studies are lacking, their favorable efficacy profile in other forms of recurrent pericarditis suggests that they may represent a valuable therapeutic alternative for selected refractory cases, particularly when conventional immunosuppressive approaches have failed [38,39].

Pericardiectomy should be reserved for highly selected patients with persistent constrictive physiology or medically refractory disease and should be performed in experienced centers. Cardiovascular MRI plays a critical role in differentiating transient inflammatory constriction from irreversible chronic constrictive pericarditis. The presence of pericardial edema or LGE supports ongoing inflammation and favors an initial trial of anti-inflammatory therapy, whereas surgical intervention is generally reserved for patients with noninflammatory or treatment-resistant constrictive disease. When surgery is required, complete pericardiectomy is preferred over partial resection because of superior long-term outcomes [40,41].

Treatment duration and additional considerations

The optimal duration of immunosuppressive therapy for lupus-associated pericarditis has not been established and is largely based on expert opinion and extrapolation from other manifestations of SLE. Azathioprine and mycophenolate mofetil are generally maintained for at least six to 12 months to reduce recurrence risk. In patients with concomitant lupus nephritis, mycophenolate-based maintenance therapy is often continued for three to five years according to current lupus management strategies. The duration of IVIG therapy should be individualized according to clinical response [42].

Restriction of vigorous physical activity is recommended during the active phase of disease and should generally continue for at least one month after clinical remission. Maintaining a heart rate below 100 bpm may reduce pericardial mechanical stress and potentially limit ongoing inflammation [43].

In patients with lupus-associated myopericarditis, therapeutic decisions should primarily target myocardial involvement because of its greater impact on morbidity, mortality, and long-term cardiovascular outcomes. High-dose glucocorticoids combined with immunosuppressive agents such as cyclophosphamide or mycophenolate mofetil constitute the cornerstone of treatment, whereas IVIG and rituximab may be considered in severe or refractory cases [44].

**Figure 2:**
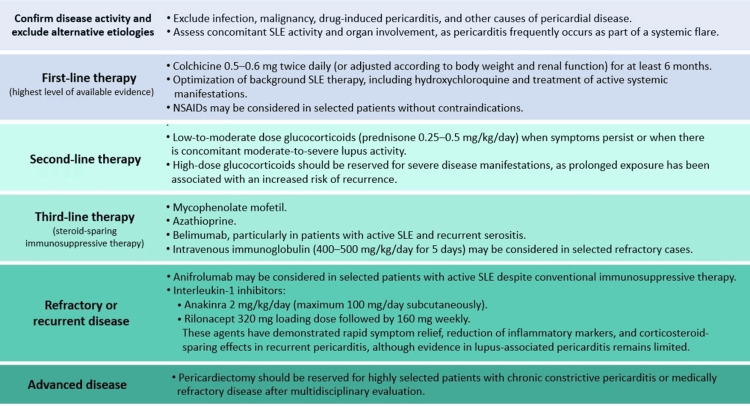
Proposed treatment algorithm for lupus-associated pericarditis NSAIDs: nonsteroidal anti-inflammatory drugs; SLE: systemic lupus erythematosus This figure was created using Microsoft PowerPoint (Microsoft Corporation, Redmond, WA, USA).

Figure 2 summarizes a stepwise treatment algorithm for lupus-associated pericarditis, emphasizing the exclusion of alternative etiologies, optimization of SLE therapy, escalation according to disease severity and recurrence, and consideration of advanced interventions for refractory cases.

## Conclusions

Pericarditis remains the most common cardiac manifestation of SLE and continues to be an important source of morbidity owing to recurrent disease and the potential development of life-threatening complications, including cardiac tamponade and constrictive pericarditis. Advances in the understanding of its immunopathogenesis, together with the increasing adoption of multimodality imaging, have significantly improved diagnostic accuracy, disease characterization, and risk stratification. Unlike idiopathic pericarditis, the management of lupus-associated pericarditis should primarily focus on achieving optimal control of the underlying autoimmune disease, as effective suppression of SLE activity frequently results in resolution of pericardial inflammation. Colchicine has emerged as a cornerstone of therapy for symptom control and recurrence prevention, whereas glucocorticoids and conventional immunosuppressive agents remain essential in selected patients with active systemic disease.

Emerging biologic therapies, including belimumab, anifrolumab, and IL-1 inhibitors, offer promising therapeutic alternatives for refractory or recurrent cases and may reshape future treatment strategies. However, the current evidence base remains limited and is derived largely from observational studies and case reports. Well-designed prospective studies are therefore needed to establish evidence-based treatment algorithms, identify optimal therapeutic sequencing, and define the duration of immunomodulatory therapy in patients with lupus-associated pericarditis.
